# *LARS2-*Perrault syndrome: a new case report and literature review

**DOI:** 10.1186/s12881-020-01028-8

**Published:** 2020-05-18

**Authors:** Maria Teresa Carminho-Rodrigues, Phillipe Klee, Sacha Laurent, Michel Guipponi, Marc Abramowicz, Hélène Cao-van, Nils Guinand, Ariane Paoloni-Giacobino

**Affiliations:** 1 Department of Genetic Medicine, University Hospitals of Geneva Rue, Gabrielle-Perret-Gentil 4, 1211 Genève 14, Switzerland; 2 Departement of Pediatrics endocrinology, Geneva University Hospital, Geneva, Switzerland; 3 Departement of ENT, Geneva University Hospital, Geneva, Switzerland

**Keywords:** Perrault syndrome, LARS2, Whole-exome sequencing, Sensorineural hearing loss

## Abstract

**Background:**

Perrault syndrome is a rare recessive and genetically heterogeneous disorder characterized by sensorineural hearing loss in males and females and gonadal dysgenesis in females. Mutations in seven different genes have been identified: *HARS2, HSD17B4, CLLP, C10orf, ERAL1, TWNK* and *LARS2*.

To date, 19 variants have been reported in 18 individuals with *LARS2*-Perrault syndrome.

**Case presentation:**

Here we describe the case of an 8-year-old girl with compound heterozygous missense mutations in the *LARS2* gene. We identified two missense mutations [c.457A > C, p.(Asn153His) and c.1565C > A, p.(Thr522Asn)] and subsequent familial segregation showed that each parent had transmitted a mutation.

**Conclusions:**

These results have implications for genetic counseling and provide insight into the functional role of *LARS2*. This case highlights the importance of an early diagnosis. Systematic genetic screening of children with hearing loss allows the early identification of a Perrault syndrome in order to ensure specific endocrinological surveillance and management to prevent secondary complications. Clinical data are compared with the other cases reported in the literature.

## Background

Perrault syndrome (MIM: 233400) is a rare recessive genetically heterogeneous disorder characterized by sensorineural hearing loss in males and females and ovarian dysfunction in females [1]. One-third of all patients have mutations in one of the seven known causative genes: *HARS2* (MIM 600783), *HSD17B4* (MIM 601860) *CLPP* (MIM 601119), *C10orf2*, (MIM606075), *ERAL1* (MIM 607435), *TWNK* (MIM 606075) and *LARS2* (MIM 604544) [2].

More recently Tracewska-Siemiatkowska A et al., 2017 described a girl with a profound congenital hearing impairment and primary amenorrhea, like in Perrault Syndrome that also present additional features like progressive retinal degeneration, agenesis of the corpus callosum, and liver disease. A homozygous variant in *YARS* (MIM 603623), a gene previously related with Charcot-Marie-Tooth disease, was identified [3].

*LARS2* encodes mitochondrial leucyl-tRNA synthetase (mtLeuRS), a 903 amino acid protein [4, 5]. Aminoacyl-tRNA synthetases then attach specific amino acids to the 3′ ends of their cognate tRNAs, which is required in the cytoplasm and mitochondria for the translation of nuclear and mitochondrial encoded genes, respectively. Apart from two aminoacyl transfer RNA synthases, glycyl-tRNA synthetase and lysyl-tRNA synthetase, all other synthetases are encoded by separate genes for nuclear and mitochondrial functions [6]. However, the human structure of mtLeuRS has not been elucidated.

Apart from hearing loss and ovarian dysgenesis in females, some patients with Perrault syndrome may present neurological symptoms, such as learning disabilities, pyramidal signs, cerebellar ataxia and motor or sensory peripheral neuropathy [7]. Here, we report the case of a young girl with bilateral severe hearing loss and bi-allellic mutations in *LARS2*.

## Case presentation

**Fig. 1:**
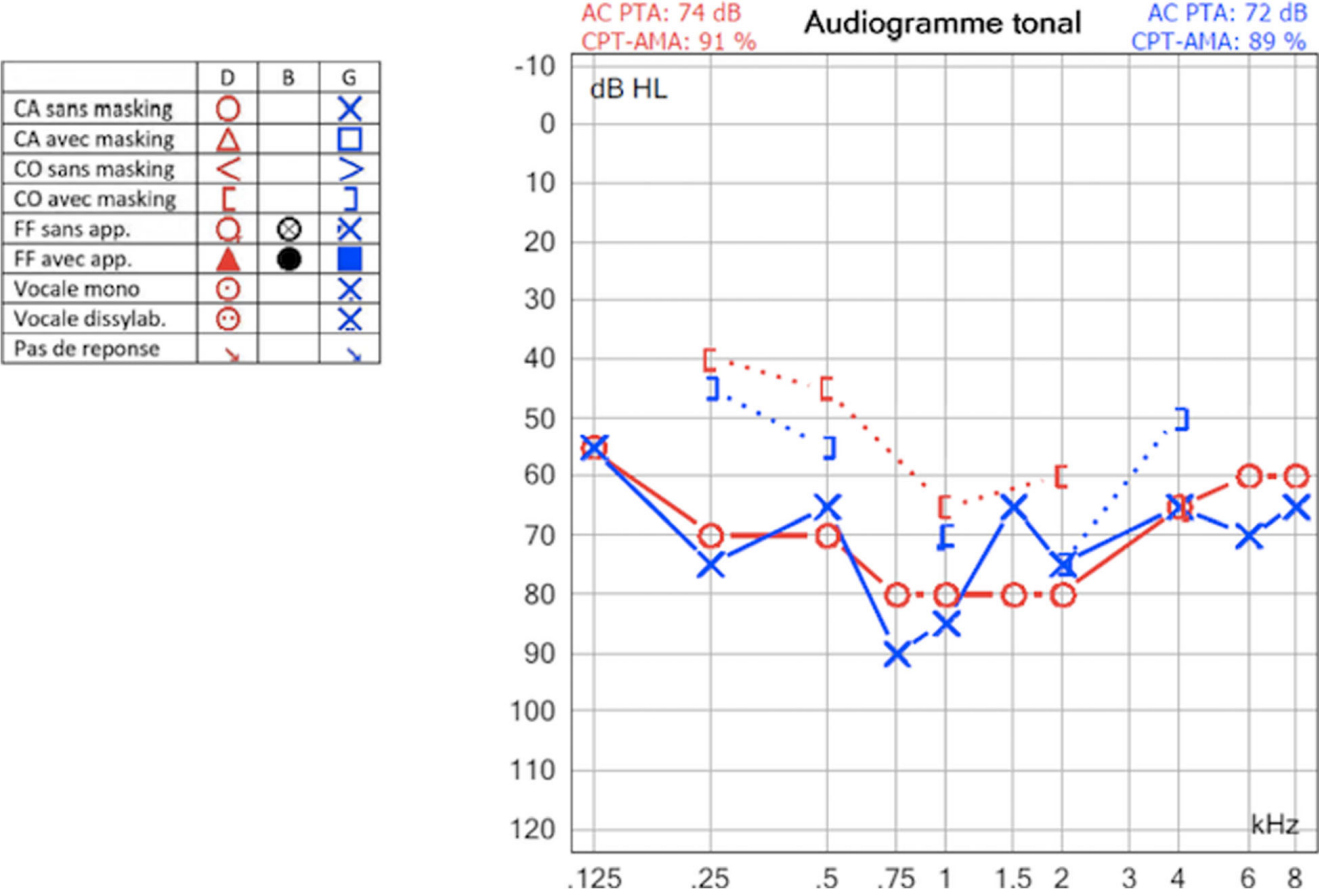
Tonal audiogram: on both sides

Our subject was an 8-year-old girl born at 36 weeks’ gestation and the first child of healthy non-consanguineous French parents. Family history was unremarkable. She has one healthy younger brother. Her birth weight was 2200 g. Newborn hearing screening was not performed at birth. Developmental milestones were normal with walking at the age of 16 months and first words at 12 months. At the age of 4 years, she started to present difficulties at nursery school that led to hearing screening. A bilateral severe sensorineural hearing loss was finally identified at the age of 7 years. Tonal audiograms (Fig. 1) did not show the upsloping pattern considered as typical for *HARS2* mutations and described in one case of a *LARS2* mutation [8]. Hearing loss was severe on both sides with a discreet U-shaped curve centered on the 1000 Hz frequency. Transient otoacoustic emission and distortion product otoacoustic emission of both ears were absent. Auditory evoked potentials showed recognized curves at 80 dB on both sides and confirmed severe hearing loss. A temporal bone computed tomography scan and magnetic resonance imaging (MRI) performed to exclude other inner ear malformations were normal.

At the age of 7 years, she received a cochlear implant on the right side (CI-522 cochlear) and responded well. One year later, the tonal free field audiogram showed 25 dB hearing levels on conversational frequencies and the vocal audiogram showed 100% of comprehension at 60 dB (intensity of normal voice). She had no neurological symptoms and her cognitive development was normal. At physical examination, she exhibited normal growth parameters and no dysmorphic features. She had astigmatism and three small café-au-lait macules. At the age of 8 years and 7 months, an endocrinological workup showed a chronological bone age that predicted a normal adult height. Hormonal assessments showed normal prepubertal values of follicle-stimulating hormone (FSH) (2.8 IU/L) and luteinizing hormone (LH) (0.7 U/L). Estradiol was below the detection limit of 17 pmol/l. Inhibin B was undetectable and the anti-Mullerian hormone (AMH) was 1.0 pmol/l. A pelvic ultrasound showed a normal prepubertal uterus measuring 32 mm in length. A right ovary measuring 16 × 6 × 6 × 6.5 mm was visualized. The left ovary was not clearly visualized. Thyroid function, insulin-like growth factor 1 and morning cortisol values (275 nmol/l) were normal. Glucose metabolism was normal with fasting blood glucose values of 4.7 mmol/l, insulin, 3.3 mIU/l, and glycated haemoglobin, 5.0% (31.0 mmol/mol).

### Molecular investigation

Written informed consent from the parents was obtained prior to molecular studies and whole-exome sequencing was performed. Exome of the patient was captured using the SureSelet QXT Human All Exon V5 kit (Agilent Technologies Inc., Santa Clara, CA, USA) and sequenced on a NextSeq500 instrument (Illumina, San Diego, CA, USA).

Reads mapping and variant calling were performed using BWA 0.7.13, Picard 2.9.0 and GATK HaplotypeCaller 3.7 and annotated with annovar 2017-07-17 and UCSC RefSeq (refGene) downloaded on 2018-08-10. The variants were searched for in various databases including dbSNP151, gnomAD 2.1, ClinVar 2018 and HGMD 2016.

Pathogenicity prediction scores were obtained for missense variants using SIFT, PolyPhen, MutationTaster and CADD. Splicing effect alterations were assessed using dbscSNV.

Our patient was found to carry two missense variants in the *LARS2* gene (NM_015340.3.3): c.457A > C, p.(Asn153His) in exon 6 rs786205560, and c.1565C > A, p.(Thr522Asn) in exon 14, rs199589947. Segregation analysis showed that the patient inherited the p.(Asn153His) variant from her father and p.(Thr522Asn) from her mother, consistent with an autosomal recessive mode of inheritance.

The c.457A > C, p.(Asn153His) variant is absent from the gnomAD database and has been classified once in ClinVar as “likely pathogenic” and once as “of uncertain clinical significance”. The amino acid change was predicted as pathogenic by all used algorithms. As the c.457A > C variant changed the second nucleotide of exon 6, it could therefore have affected splicing, as predicted by the dbscSNV (score = 0.93) and recently reported in homozygosity by Al-Jaroudi et al. [9] The c.1565C > A, p.(Thr522Asn) variant is present in the gnomAD database (MAF = 2.8 × 10^− 4^) at a frequency compatible with a recessive mode of inheritance. It is also predicted as pathogenic by all used algorithms and is classified three times as “pathogenic” and once as “likely pathogenic” in ClinVar. It has already been reported in the literature as associated with Perrault syndrome [8, 10]. According to the ACMG guidelines [11, 12], we classified the p.(Asn153His) and p.(Thr522Asn) variants as pathogenic. The presence of these two variants in compound heterozygosity indicated that our patient presented a Perrault syndrome.

## Discussion and conclusions

We described a young girl with a Perrault syndrome caused by a bi-allelic *LARS2* mutations. To our knowledge, our patient is only the second case of a p.Asn153His Perrault syndrome reported in the literature and the first case reported in compound heterozygosity. The first case reported with p.Asn153His in homozygosity was a 27- year-old female with Perrault syndrome type 1 [9]. Regarding the second mutation, p.Thr522Asn, has been already described several times. It has also been implicated in a *LARS2* more severe phenotype described by Riley et al. [13] They described a lethal, multisystem metabolic disorder characterized by severe lactic acidosis, hydrops and sideroblastic anemia, impaired cardiac function, disordered coagulation, pulmonary hypertension and progressive renal disease associated with the bi-allelic *LARS2* p.Thr522Asn and p.Ala430Val mutations [7, 11]. Thus, the presence of p.Thr522Asn in our patient reinforced the idea that an association with the p.Ala430Val variant would have been more likely to be more damaging than p.Thr522Asn already described in homozygosity in Perrault syndrome [8, 10].

The identification of two missense variants both previously reported in patients with Perrault syndrome is in full agreement with all previous variants being of missense type, and it has been proposed that inactivating variants in these essential genes, may not be compatible with life.

To date, 19 variants have been identified in *LARS2* in 18 individuals with Perrault syndrome (Table 1). A comparison of the literature showed that bilateral hearing loss was highly variable, ranging from moderate to profound, and sometimes progressive. Of note, all individuals with a p.Thr522Asn Perrault syndrome, either homozygous or compound heterozygous, presented with sensorineural hearing loss, which was worse at low frequencies [5, 10]. Age of onset also varied between 18 months and 32 years, but it was difficult to evaluate as detailed information was not always available.

**Table 1:** (adapted fom Kosaki et al. 2017): ID, identity of the patient; CI, cochlear implant; LF, low frequency; NA, not applicable; NR, not recorded: POI, primary ovarian insufeciency; SNHL, sensorineural hearing loss; FSH, follicular stimulating hormone; LH, luteininsing hormone.

ID	Family 1, II-1	Family 1, II-2	Family 1, II-3	Family 2, proband	II1	II3 proband	P2:II-1 proband	P2:II-2	P3:II-1 proband	P3:II-2	Patient III-3	III-1 proband	III-5	Patient 1 - proband	Patient 2	Patient 1	Patient 2	Patient 3	Patient 4	Index case	Our Patient
**Variants**	c.1565C>A (p.Thr522Asn)	c.1565C>A (p.Thr522Asn)	c.1565C>A (p.Thr522Asn)	c.1077delT (p.IIe360PhefsTer15) c.1886C>T (p.Thr629Met)	c.899C>T (p.Thr300Met) c.1912G>A (p.Glu638Lys)	c.899C>T (p.Thr300Met) c.1912G>A (p.Glu638Lys)	c.1565C>A (p.Thr522Asn)	c.1565C>A (p.Thr522Asn)	c.351G>C (p.Met117IIe) c.1565C>A (p.Thr522Asn)	c.351G>C (p.Met117IIe) c.1565C>A (p.Thr522Asn)	c.1358G>A (p.Arg453Gln) c.1886C>T (p.Thr639Met)	c.1565C>A (p.Thr522Asn)	c.1565C>A (p.Thr522Asn)	c.880G>A, p.(Glu294Lys); c.1556C>T, p.(Thr519Met)	c.880G>A, p.(Glu294Lys); c.1556C>T, p.(Thr519Met)	c.462delT, p.(Lys155Asnfs*3); c.1120A>C, p.(Ile374Leu)	c.1987C>T, p.(Arg663Trp); c.371A>T, p.(Asn124Ile)	c.516G>T, p.(Arg172Ser); c.1028C>T, p.(Thr343Met)	c.683G>A, p.(Arg228His); c.880G>A, p.(Glu294Lys)	c.457A>C, p.(Asn153His)	c.457A>C, p.(Asn153His); c.1565C>A, p.(Thr522Asn)
**References**	Pierce et al. (2013) [8]	Pierce et al. (2013) [8]	Pierce et al. (2013) [8]	Pierce et al. (2013) [8]	Solda et al. (2016) [14]	Solda et al. (2016) [14]	Demain et al. (2017) [10]	Demain et al. (2017) [10]	Demain et al. (2017) [10]	Demain et al. (2017) [10]	Lerat et al. (2016) [16]	Zerkaoui et al. 2017 [5]	Zerkaoui et al. 2017 [5]	Kosaki et al. (2018) [7]	Kosaki et al. (2018) [7]	Van der Knaap et al. (2019) [15]	Van der Knaap et al. (2019) [15]	Van der Knaap et al. (2019) [15]	Van der Knaap et al. (2019) [15]	Al-Jaroudi et al. (2019) [9]	our case
**Ethnicity**	Palestinian	Palestinian	Palestinian	Slovenian	Italian	Italian	Argentinian	Argentinian	White British	White British	Sri Lankan	Marrocan	Marrocan	NR	NR	NR	NR	NR	NR	Saudi arabia	France
**Consanguinity**	Yes	Yes	Yes	No	No	No	No	No	No	No	No	Yes	Yes	No	No	NR	NR	NR	NR	Yes	No
**Sex**	Male	Female	Male	Female	Male	Female	Female	Male	Female	Male	Female	Female	Male	Female	Female	Female	Male	Male	Female	Female	Female
**Age at last assessment (years)**	17	17	13	30	40	31	27	26	25	26	NR	23	16	17	11	32	37	8	45	27	8
**Sensorineural hearing loss**																					
**Age at diagnosis (years)**	3-5	3-5	3-5	3-5	congenital	-	8	26	2.5	2.5	<3	23	16	18 m.o.	congenital	congenital	congenital	congenital	congenital	congenital	4
**Degree of hearing loss**	Severe to profound	Right : severe at low frequencies, moderate at high frequencies. Left: moderate at low frequencies, mild at high frequencies	Severe to moderate at low frequencies, moderate to mild at high frequenies	Severe	Profound	Profound	Moderate	Mild/ moderate	Severe/ profound	Severe/ profound	Moderate	Moderate/ profound	Moderate/ profound	NR	NR	Profound	NR	Profound	NR	Profound	Severe
**Notes**	Bilateral LF SNHL	LF SNHL	Bilateral LF SNHL	None	Progressive SNHL	Progressive SNHL	LF	LF	LF	LF	Not progressive	Progressive	Progressive	-	-	-	-	-	-	-	Bilateral SNHL
**Intervention**	NR	No hearing aid	NR	NR	Bilatera CI	Bilatera CI	CI	NR	CI	NR	NR	Hearing aid	Hearing aid	NR	NR	NR	Hearing aid	Unilateral CI	Unilateral CI	NR	Unilateral CI
**Pelvic US**	NA	Small uterus, ovaries not visualized	NA	NR	NA	Bicornate uterus, hypoplastic left ovary, right ovary not visualised	Small uterus and ovaries	NA	Small uterus and ovaries	NA	NR	Small uterus, ovaries not visualized	NA	Hypotrophic uterus, ovaries not visualized	Hypotrophic uterus, ovaries not visualized		NA	NA	Streak ovaries	Hypoplastic uterus and streak ovaries	Left ovary not well visualised
**Menarche**	NA	No	NA	Yes	NA	Yes	No	NA	Yes	NA	No	NR	NA	No	No	Yes	NA	NA	Yes- 16	No	No
**POI- age if menarche achieved**	NA	Yes	NA	Yes-19	NA	Yes-28	NA	NA	Yes	NA	NA	NA	NA	NA	NA	Yes- 29	-	-	Yes- soon after menarche	-	NA
**FSH (IU/I)**	NR	76.9	NR	101	NR	118	99.6 (2.3-29)	NR	74 (<30)	3.1 (1-11)	NR	51	NR	46.90 IU/L	22.06 IU/L	NR	NR	NR	NR	88.4 IU/L	NR
**LH (IU/I)**	NR	30.3	NR	NR	NR	45.4	48.0 (1.7-52)	NR	63 (<30)	3.9 (1-11)	NR	16.29	NR	9.95 IU/L	3.04 IU/L	increased	NR	NR	increased	31.4 IU/L	NR
**Estradiol**	NA	NR	NA	NR	NA	NR	7.04 (10-388) pg/ml	NA	91 (>180) pmol/l	NA	NR	NR	NA	<10 pg/mL	<10 pg/mL	decreased	NR	NR	decreased	213 pmol/ L	NR
**Neurological features**	No	No	No	No	No	No	No	No	No	No	No	No	No	Developmental delay; behavioural problems; ataxic gait	Learning difficulties	Cerebellar ataxia; spasticity; swallowing difficulties	Hypotonia at birth; autistic behaviour; hyperactivity; aggression; atypical seizures; extrapyramidal dysfunction	Hypotonia at birth; hyperkinesia; self-mutilation; temper tantrums; aggression; mild pyramidal signs with brisk reflexes but no ataxia.	Pyramidal dysfunction; axial ataxia	No	No
**Additional features**	No	No	No	No	No	No	No	No	Mild facial dysmorphia, hemidystrophy	Hypopadias, mild facial dysmorphia, normal testosterone	Cleft palate	Marfanoid habitus	Marfanoid habitus	Obesity; strabismus; osteoporosis; fatty liver; scoliosis			Macrocephaly; inguinal hernia; MRI showed early-onset vascular abnormalities		MRI showed early-onset vascular abnormalities	Marfanoid habitus; tarlov cysts; degenerative changes of the vertebral column	Astigmatism; 3 café-au-lait macules

Amenorrhea was most frequently primary and there appeared to be no obvious genotype-phenotype correlation [15].

It is still not clear if *LARS2* can be responsible for an isolated hearing loss in the case of our young female patient and other prepubertal female patients described before. Follow up is essential to elucidate this question.

Neurological symptoms were present in five families with neurodevelopmental delay, gait ataxia, behavioral problems and pyramidal dysfunction [7, 15]. Van der Knaap et al. [15] reported three patients who developed severe neurological features in later life. Cerebral MRIs performed at adult age showed extensive white matter abnormalities and additional signs of early-onset vascular abnormalities were observed in two patients [13]. However, as most patients were relatively young at genetic diagnosis, it remains unclear whether neurological symptoms may develop later [5].

In our patient, the fact that the diagnosis was made at an early age was crucial for this young girl. She was referred to the pediatric endocrinologist for evaluation and follow- up. Endocrinologic work-up showed normal prepubertal values for FSH, LH and estradiol. A pelvic ultrasound showed a normal prepubertal uterus and allowed visualization of a right gonad. Visualization of the left gonad was difficult. Inhibin B and AMH were measured at a time point where both hormones are naturally low: inhibin B was undetectable and AMH was below normal values. While visualization of the gonads is reassuring, a follow-up is necessary to determine the ovarian function of the patient. Thyroid function and glucose metabolism were analyzed in the context of previous reports of abnormalities of these parameters [16] and proved normal.

Pubertal development will be monitored in the future in order to induce puberty and permit normal bone mineralization. Should ovarian insufficiency be confirmed, oocyte cryopreservation should be considered. Hearing loss should be assessed and treated by a multidisciplinary team including an audiologist and otolaryngologist. Possible interventions for those with hearing loss include special educational resources, hearing aids, vibrotactile devices and cochlear implantation, which is an option for children older than 12 months with severe-to-profound hearing loss [17]. In addition, due to highly intra- and inter-clinical variably, we tested her younger brother to exclude the presence of familial mutations and hence, the risk of hearing loss.

More recently, leukodystrophy was also associated with *LARS2* pathogenic variants [15]. Concerning the development of neurological problems, systematic neurological surveillance is still not indicated, but it should be kept in mind that these may eventually develop later in life. More reports of older patients will help to elucidate the eventual progression of this syndrome.

Our case demonstrates that whole-exome sequencing is essential in the diagnosis of hearing loss in children. Benefits of receiving an early genetic diagnosis include the provision of prognostic information, streamlined care, accurate recurrence risk advice and, where appropriate, screening and treatment for associated health conditions.

## Data Availability

All data generated or analyzed during this study are included in this published article. The mutations described in the current study are available in the ClinVar repository [ID: 55871 and 191173] https://www.ncbi.nlm.nih.gov/clinvar/variation/191173/ and https://www.ncbi.nlm.nih.gov/clinvar/variation/55871/ . The complete sequence datasets generated during the current study are not publicly available because individual privacy could be compromised.

## Abbreviations

FSH: Follicle stimulating hormone
LH: Luteinizing hormone
AMH: Anti-Müllerian Hormone
